# Gene microarray analysis revealed a potential crucial gene RACK1 in oral squamous cell carcinoma (OSCC)

**DOI:** 10.1080/19768354.2018.1443493

**Published:** 2018-02-27

**Authors:** Jian-Wei Zheng, Yinshen Yang, Shujuan Yang, Wei Zhou, Hongtian Qiu, Xiaoping Li, Qiuyun Cai, Ting Li, Gang Luo

**Affiliations:** a Shenzhen Stomatological Hospital, Southern Medical University, Shenzhen, People’s Republic of China; b Key Laboratory of Oral Medicine, Guangzhou Institute of Oral Disease, Stomatology Hospital of Guangzhou Medical University, Guangzhou, People’s Republic of China; c Department of Public Health and Social and Behavioral Sciences, West China School of Public Health, Sichuan University, Chengdu, People’s Republic of China

**Keywords:** RACK1, OSCC, gene microarray, gene profile

## Abstract

Oral squamous cell carcinoma (OSCC) is the sixth most common cancer worldwide, which appears as a consequence of multiple molecular genetic events in various chromosomes and genes. In order to unveil the possible mechanisms underlying OSCC tumorigenesis, the OSCC-related gene expression variance and the gene interaction network should be further investigated. Herein, we conducted the NimbleGen Human Gene Expression Microarray to analyze expression heterogeneity between OSCC primary tumor tissue and its adjacent normal tissue from two patients. A total number of 7872 out of 32,448 detected genes are differentially expressed in OSCC. Gene ontology (GO) analysis demonstrated that these differentially expressed transcripts were critical in a series of metabolic processes, cancer-related signal pathways, and biological regulations. KEGG signaling pathway enrichment suggested a number of pathways (metabolic process and immune response) which are frequently enrolled during cancer progression. 15 most differential regulated genes between OSCC tumor and non-tumor were confirmed by quantitative reverse transcription polymerase chain reaction (qRT-PCR). Furthermore, the interaction network analysis of these confirmed genes by STRING database showed the two subunits of RACK1 had direct interaction with 14 differential proteins. This bioinformatics research lends support about the critical role of RACK1 which functions as a key node protein driving OSCC development.

## Introduction

Oral squamous cell carcinoma (OSCC) is among the most lethal malignancies with 30,000 new cases diagnosed and approximately 11,000 deaths every year (Chaturvedi et al. 2008; Wang et al. 2013). Early-to-moderate-stage OSCC (American Joint Committee on Cancer stages I-III) is often treated surgically, with radiotherapy given in the presence or absence of chemotherapy in the post-operative adjuvant setting for high-risk patients(Lo et al. 1976). In advanced (stage IV) cases, multidisciplinary non-surgical approaches are being applied with increasing frequency to improve disease control, prolong survival, and maintain life quality for patients. Even when the appropriate combination of surgical and non-surgical approaches is used, more than half of patients still experience cancer recurrence (Massano et al. 2006). Furthermore, recurrent and distantly metastatic OSCC carry particularly poor prognosis(Johnson et al. 1992). Therefore, it is necessary to conduct studies on OSCC mechanisms to develop more effective and efficient therapies.

Like other tumor types, the OSCC occurrence is regarded as an outcome of multiple factors (Anneroth et al. 1987; Miller and Johnstone 2001; Chiou et al. 2008) and a complicated process involved in the network among multifarious genes and proteins (Hu et al. 1991; Xia et al. 1999). Currently, most of OSCC researches mainly focus on the critical role of a single gene or protein during tumor deterioration, **such as oncoprotein EZH2 (**Shiogama et al. 2013**) and histone modifier hMOF (**Li et al. 2015**).** However, to well understand the mechanism of OSCC tumorigenesis, specific attention should be paid to the difference of signal network channel protein and OSCC-related genes instead of a single gene or protein, and the interaction of these proteins also deserves diligent evaluation.

DNA microarray (also commonly known as DNA chip or biochip) is a collection of microscopic DNA spots attached to a solid surface, which is often used to measure the expression levels of a batch of genes simultaneously or genotype of multiple genomic regions (Schena et al. 1995; Eisen et al. 1998; Golub et al. 1999; Tusher et al. 2001). The pre-designed oligonucleotides are densely and orderly arranged to form a chip. With fluorescence dye samples (such as Cy3, Cy5 etc.) labeling the probe, oligonucleotides on the chip interact with the probe according to the principle of base pairing (DeRisi et al. 1997). The result then can be detected and collected by various means such as confocal laser scanning and fluorescence signal acquisition. The obtained sequence information is finally analyzed by bioinformatics interpretation (Kononen et al. 1998; Olsen et al. 2010). Computational technologies are used to accelerate or fully automate the process, quantification and analysis of massive highly informed biomedical imagery. DNA microarray is able to analyze the sequence and function of genes in a high through output at the same time.

In this study, the DNA microarray is applied in analyzing the difference of gene expression between OSCC and adjacent normal tissue. The gene distribution and characteristic of the differentially expressed gene patterns were analyzed by the GO analysis, indicating that these deregulated genes were involved in a series of metabolic processes, cancer-related signal pathways, and molecular functions. Furthermore, we have also selected out the representative 15 differently expressed genes with the consistent protein expression profile proved by our previously report. We have further demonstrated that these gene expressions are significantly different between OSCC tumor and non-tumor by qRT-PCR. The interaction network of these selected genes and proteins was built in the STRING database, which revealed the crucial role of the receptor for activated C kinase 1 (RACK1) as the core protein of the entire structural network in OSCC.

## Materials and methods

### Patient samples collection

All experiments were preceded according to hospital regulations and medical ethics standards of School and Hospital of Stomatology, Guangzhou Medical University, Guangzhou, China. Two excisions of OSCC and peripheral normal tissues (2 cm above) were collected by surgery in Stomatological Hospital of Guangdong Province in 2013. None of the patients received chemotherapy or radiotherapy before surgery. Informed consents were obtained and the study was approved by School and Hospital of Stomatology, Guangzhou Medical University ethical committee, Guangzhou, China **(Guangzhou medical university: no.2016-067)**.

### DNA microarray and bioinformatics analysis

The total RNA of tumor and surrounding normal tissues were collected using Trizol. One microgram of total RNA was transcribed into cDNA using RevertAid™ H Minus First Strand cDNA Synthesis Kit. 1 μg cDNA was incubated with Random 9-mer labeled by Cy3. 3 μL resulting cDNA labeled with Cy3 was incubated with 8.7 μL Master Mix at 42°C for 5 min, then another 6 μL above mixture was added into the hybridization kit with the gene chip at 42°C for 16 h. The NimbleGen Human Gene Expression Microarrays (Microarrays version: Roche NimbleGen Homo sapiens 12 × 135 k) were used for the differential gene expression between tumor and normal tissues. The gene chip was scanned by Axon GenePix 4000B microarray scanner, with NimbleScan (version 2.5) software for the image analysis that converts the image signal to digital signal. Gene expression microarray data was analyzed by Agilent GeneSpring GX v12.0.

### Quantitative real-time PCR

32 excisions of OSCC and neighboring tissues (2 cm above) of equal quantity were gathered by surgery from patients without chemo- or radiotherapy in Stomatological Hospital of Guangdong Province in 2013, with their awareness and consent alongside the approval of local ethical committee. The total RNA of these samples were obtained using Trizol, in which 1 µg of them was transcribed into cDNA using RevertAid™ H Minus First Strand cDNA Synthesis Kit. To assess mRNA levels, qRT-PCR was performed using FastStart Universal Probe Master with forward and reverse primers listed below. mRNA levels were normalized against the housekeeping gene GAPDH using forward 5′-ATCAAGAAGGTGGTGAAGCAGGCA-3′ and reverse 5′-TGGAAGAGTGGGAGTTGCTGTTGA-3′ primers. PCR parameters are as follows: 60 s of Taq activation at 95°C, followed by 40 cycles of PCR at 95°C × 20 s, and 1 cycle of 95°C × 15 s, 57°C × 60 s and 95°C × 15 s. The gene primers detected are listed in Table 1.

**Table 1.** RT-PCR primers of 15 selected genes.

Accession number	Gene	Forward / Reverse	Primers
NM_006098	RACK1	Forward	CTCTGGGATCTCACAACGGG
		Reverse	TGCACACACCCAGGGTATTC
NM_002964	S100A8	Forward	AGCCCTGCATGTCTCTTGTC
		Reverse	ACGTCTGCACCCTTTTTCCT
NM_002965	S100A9	Forward	CCCACGAGAAGATGCACGAG
		Reverse	CCTCCTGATTAGTGGCTGTGG
BC034687	S100A7	Forward	GATTGACAAGCCAAGCCTGC
		Reverse	GGCTATGTCTCCCAGCAAGG
BC009200	GDI-beta	Forward	ATGAATGCATCCTCCCCCTTT
		Reverse	ACTCATTGGGCCAGCAACAA
NM_000700	ANXA1	Forward	CGAAACAATGCACAGCGTCA
		Reverse	TCCTCAGATCGGTCACCCTT
BC001388	ANXAA2	Forward	GGACGCGAGATAAGGTCCTG
		Reverse	GCTTTCTGGTAGTCGCCCTT
NM_001154	ANXA5	Forward	AGGGTACTACCAGCGGATGT
		Reverse	AGTCTCGCGGTCAATGGTTT
NM_000100	CST B	Forward	ATTCAAGAGCCAGGTGGTCG
		Reverse	AAGTGCACGCTCTGGTAGAC
NM_001885	CRYAB	Forward	CCGGCAAAGAGCAGGTATCA
		Reverse	GACTCCAACAGGTGCTCTCC
NM_005563	STMN1	Forward	TTGGTGCTCAGAGTGTGGTC
		Reverse	GGGGAAAGGGGGAATTCTGG
NM_002307	LGALS7	Forward	GAGAATTCGCGGCTTGGTTC
		Reverse	CCAGCCGGGGGTTGAAAT
AY129319	EIF5A	Forward	ACCCCTTTAGATGGGGACC
		Reverse	TTAACAAATGTGTCGGGGAGA
NM_003186	TAGLN	Forward	GAAGCCTTCTTTCCCCAGACA
		Reverse	ATCACGCCATTCTTCAGCCA
NM_006919	SERPINB3	Forward	GCTGAAGATCGCCAACAAGC
		Reverse	CCAATGTGGTATTGCTGCCA

### GO analysis and KEGG pathway analysis

The Gene Ontology (http://www.geneontology.org) was performed to clarify the functional distribution of the differentially expressed genes in OSCC, which mainly covers in three particular parts: Biological Process, Cellular Component, and Molecular Function. We applied Fisher’s exact test to calculate the *p*-value with a 0.05 cutoff. Pathway analysis is performed by mapping genes to KEGG pathways database. Fisher'’s exact test to calculate the *p*-value with a 0.05 cutoff.

### Construction of relationship among differential genes

Among numerous protein interaction databases, STRING database 9 was employed to guide the search for epistasis. STRING is among the largest databases of known and predicted protein–protein interaction which integrates reported interactions from dedicated interaction databases and multipurpose ones centered on specific model organisms. The interactions inside STRING consist of direct (physical) and indirect (functional) ones that derived from genomic context, high throughput, co-expression along with previous knowledge. While no distinction has been made among different types of interactions, the confidence of these interactions has been well differentiated – each protein–protein interaction in STRING has a confidence score. We only focused on the high-scored genes (i.e. interactions with a score over 0.7) in autosomal chromosomes and finally screened out 15 genes. Thereafter, these 15 genes were input into STRING (http://string.embl.de) to analyze and construct the relationship network by means of bioinformatics as well as to find the potential relationship among protein subunits.

## Results

### DNA microarray of patient-derived OSCC and the tumor side tissue

**Figure 1.**
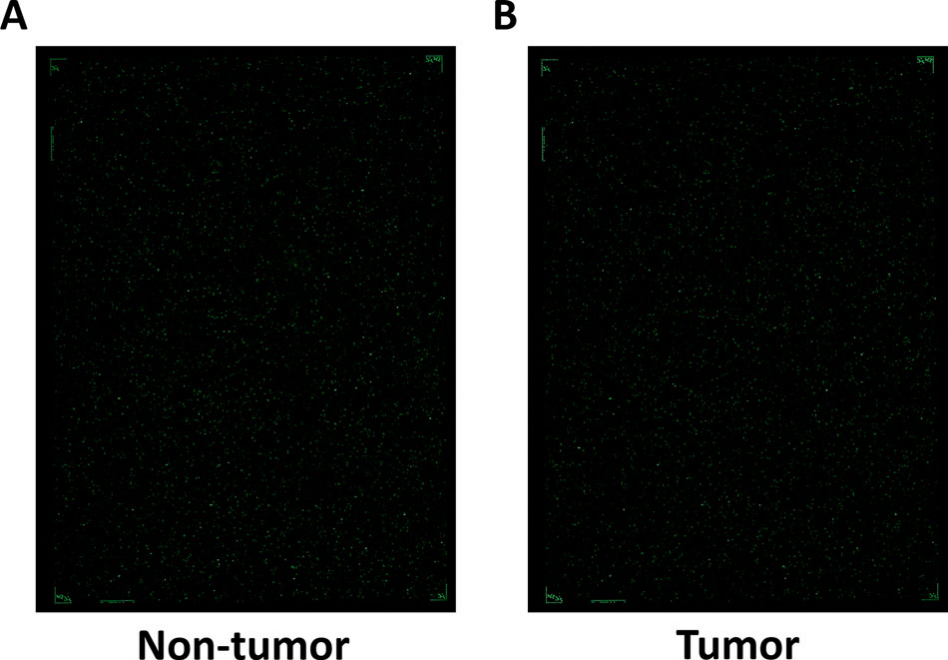
The scanning results of gene microarray. A, adjacent normal tissue; B, OSCC tumor primary tissue.

Gene expressions of OSCC and adjacent non-tumor samples nearby from two patients were detected by microarray chips. The fluorescence signal shown in Figure 1 demonstrated a high signal intensity and uniform chip hybridization, clear and balanced gene point, indicating the fairly ideal outcome.

**Figure 2.**
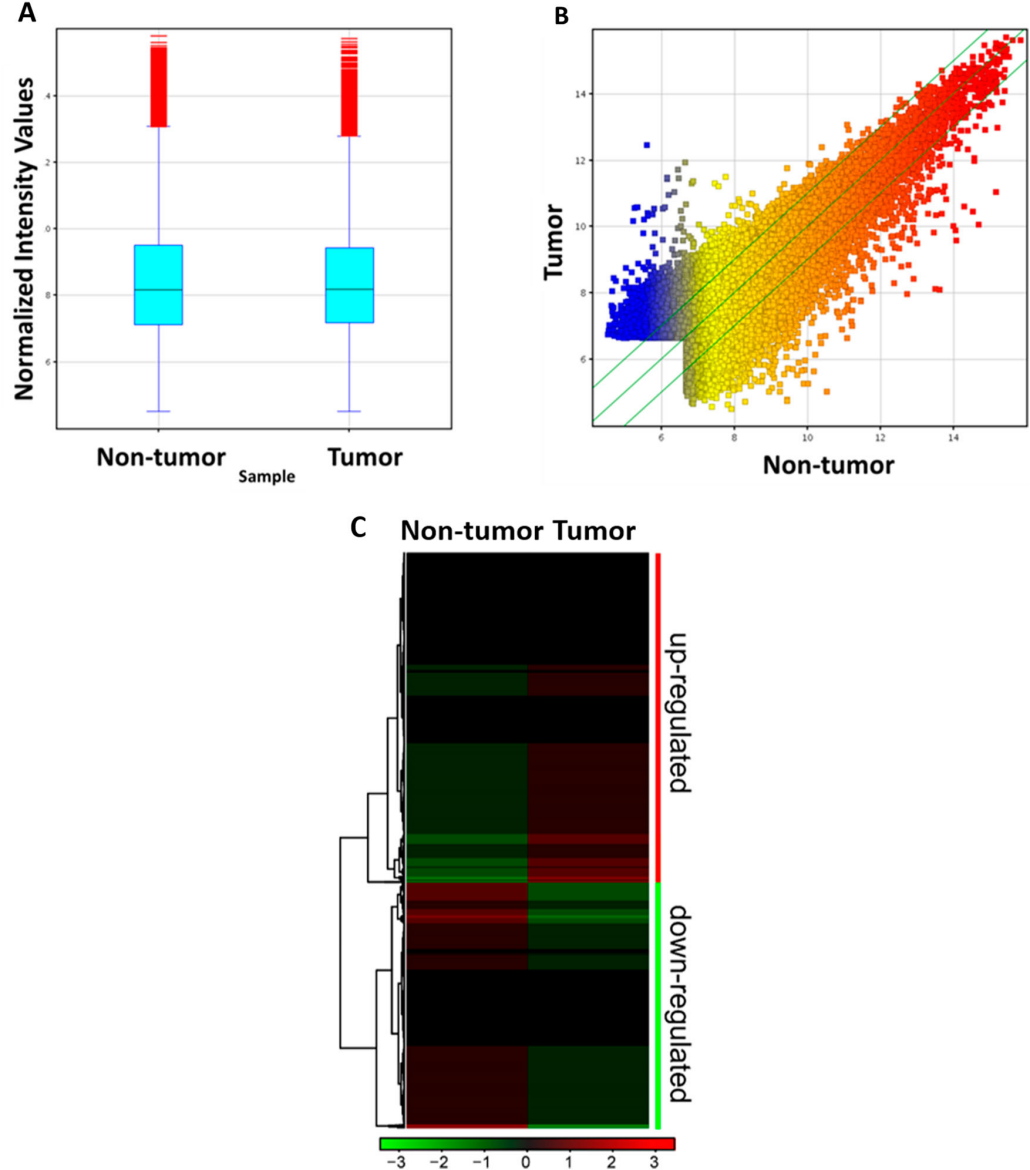
The gene expression pattern of OSCC tumor and non-tumor. A, Box-plot to show the distribution of detected mRNAs between OSCC non-tumor and tumor; B, scatter plot to exhibit the expression variance between OSCC non-tumor and tumor; *X* and *Y* values represent the the averaged normalized microarray signals (log2). The green lines represent  ±  2 fold-changes; C, Heatmap of differential expressed genes between OSCC non-tumor and tumor. The colors show the relative gene expression levels with downregulation represented by shades of green and upregulation by shades represented by red.

Further analysis of the gene microarray data was assessed and exhibited by Box-Plot (Figure 2(A)), Scatter Plot (Figure 2(B)) and the Hierarchical Clustering (Figure 2(C)). Figure 2(A) showed that the log_2_ rates of all samples are similar without significant difference, which is suitable for further data analysis. The Scatter Plot was employed to evaluate the difference of the gene expression between two microarrays. The *X/Y* axes represent the standard signal values of control and test group (log_2_); the green lines are folding change lines. As displayed in Figure 2(B), most genes are located between the upper and lower green folding lines, which suggest that the majority of the detected genes remain almost unchanged during OSCC development. However, there are still some dots positioned far away from the lines, showing the presence of the potential tumor-related genes between OSCC and the adjacent tissue. We further applied hierarchical clustering and got similar results to Figure 2(B). The red regions indicate relatively highly expressed genes while the green means lowly expressed ones, like in Figure 2(C), the conspicuous difference is shown between the two samples. According to the screening criteria of the differential genes, a total number of 7872 out of 32,448 tested genes are differential expression genes of OSCC, which accounts for 24% of the total. Among them, 3800 genes are up-regulated genes, and 4072 are down-regulated ones.

### Characterization of differential gene profile in OSCC

**Figure 3.**
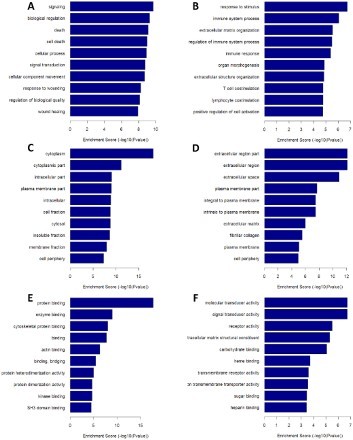
The GO analysis of the deregulated repressed and induced gene profile. A, down-regulated genes ruling biological process; B, up-regulated genes in charge of biological process; C, down-regulated genes controlling cellular components; D, up-regulated genes responsible for cellular components; E, down-regulated genes dominating the molecular functions; F, up-regulated genes directing molecular functions.

GO analysis was carried out to find out the functional distribution of the differential genes. Notably, we found these genes are involved with some critical processes during cancer initiation and progression. Functions of these genes distribute on three major components: biological process, cellular component, and molecular function. The 715 down-regulated genes responsible were enriched with some cell death signaling and wound healing, death (9.12), cell death (8.98), response to wounding (8.24), and wound healing (7.90) (Figure 3(A)). Coordinately, the 532 up-regulated genes in charge of biological process (Figure 3(B)) were discovered by functional enrichment analysis. These genes are mainly concentrated in immune-regulation, such as immune system process (6.06), regulation of immune system process (5.49), immune response (5.39), lymphocyte costimulation (4.77), T cell co-stimulation (4.77). In regarding to the cellular component distribution, the 149 subhorizon down-regulated genes were chiefly enriched in cytoplasm parts 18.17 cytoplasm and 11.18 cytoplasmic parts (Figure 3(C)), while the 41 up-regulated genes mainly related with extracellular regions, including extracellular region part (12.14), extracellular regions (12.14) and extracellular space (10.91) (Figure 3(D)). With molecular function GO analysis, 132 down-regulated genes were figured out to be associated with different protein–protein binding interactions, such as protein binding (17.68), enzyme binding (8.97), cytoskeletal protein binding (8.00), (Figure 3(E)). But the 97 up-regulated genes were uncovered to be involved with signal transducer activities (6.74) and molecular transducer activities (6.74) (Figure 3(F)).

**Figure 4.**
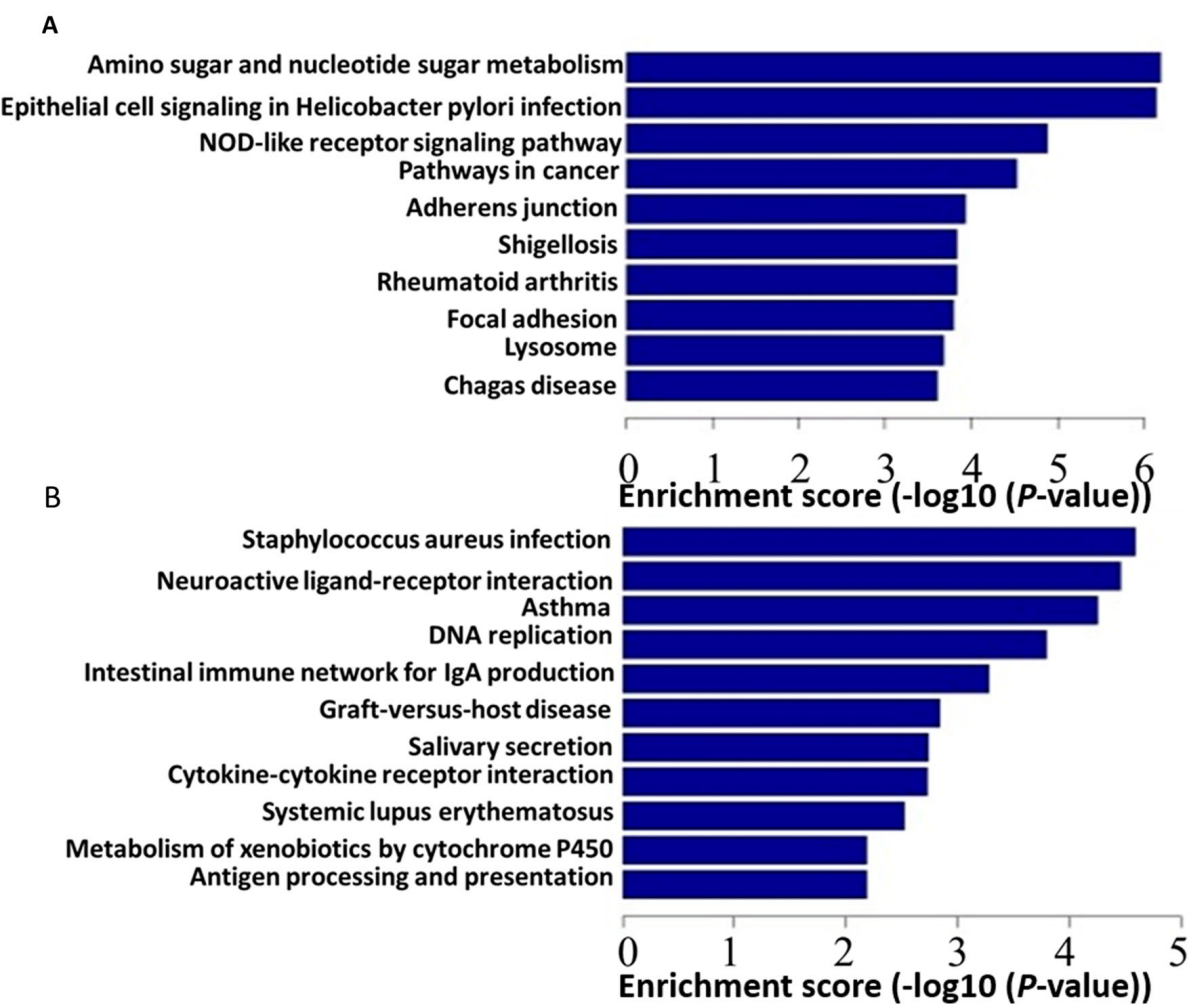
The enriched signaling pathways of differentially expressed genes of OSCC. A, down-regulated genes related signaling pathways; B, up-regulated genes related signaling pathways. Those aberrantly expressed genes were enriched into various signaling pathways based on KEGG database. The X axis represents enrichment score and Y axis represents the specific pathways.

To further explore the gene profile, we applied KEGG pathway database to examine the pathway distribution of these aberrantly expressed genes (Figure 4). Repressed genes were found in 55 biological pathways, which are concentrated in pathways of metabolic (sugar metabolism (6.19)), immune-related (NOD-like receptor signaling pathway (4.88)),oncogenic (Pathways in cancer (4.52))and adhesive (Adherens junction (human)(3.93)) pathways (Figure 4(A)). The biological pathways of the up-regulated genes were found in 26 pathways, which are mainly enriched in tumor proliferation and immune related pathways, such as DNA replication(3.80),intestinal immune network(3.28),and cytokine-cytokine receptor interaction(2.72) (Figure 4(B)). We found these differentially expressed genes contribute to a complicated network of many signal pathways involving in OSCC carcinogenesis.

### qRT-PCR verification of differential expression genes in OSCC and tissues nearby

By integrating our gene expression data and previous protein expression screening result, we have finally screened out 15 most differentially expressed gene. These genes expression was further confirmed by qRT-PCR among 32 paired OSCC tissues. In consistence, all the differential genes verified among 32 tissues have statistical differences (*P* < 0.001), reconfirming the reliability of gene expression microarrays.As shown in Table 2, among these 15 differential genes, RACK1, ARHGDIB, STMN1, LGALS7, EIF5A, and TAGLN were significantly overexpressed in OSCC tumor compared with non-tumor. On the other hand, 9 of them are significantly down-regulated, including S100A7, S100A8, S100A9, ANXA1, ANXA2, ANXA5, CSTB, CRYAB, and SERPINB3.

**Table 2.** RT-PCR analysis of OSCC and neighboring tissue.

Gene	Regulation	Log2 (fold changes)	*p*
RACK1	Up	1.35	<0.001
S100A8	Down	−1.35	<0.001
S100A9	Down	−1.41	<0.001
S100A7	Down	−1.33	<0.001
ARHGDIB	Up	0.95	<0.001
ANXA1	Down	−1.55	<0.001
ANXA2	Down	−1.80	<0.001
ANXA5	Down	−1.50	<0.001
CSTB	Down	−1.31	<0.001
CRYAB	Down	−1.87	<0.001
STMN1	Up	1.53	<0.001
LGALS7	Up	1.43	<0.001
EIF5A	Up	2.03	<0.001
TAGLN	Up	1.61	<0.001
SERPINB3	Down	−1.84	<0.001

### Construction of relationship among the differential genes

**Figure 5.**
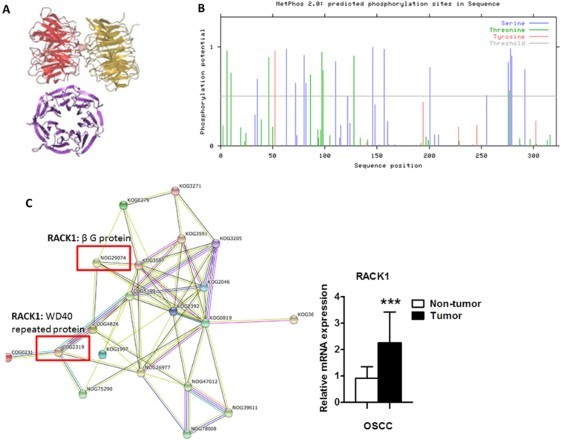
RACK1 as a key center in the gene-gene interaction network among differential expressed proteins of OSCC. A, 3D structure of RACK1; B, phosphorylation sites prediction of RACK1; C, gene-gene interaction network of 15 degregulated genes in OSCC. COG4826(SERPINB3), COG2319(RACK1), KOG1997(STMN1), KOG2392(SERPINB3), KOG3587(LGALS7), KOG2046(TAGLN), KOG3205(ARHGDIB), KOG3591(CRYAB), KOG0819(ANXA1, 2, 5), NOG26977(ANXA2), NOG26977(ANXA2), COG5199(TAGLN), KOG1997(STMN1), KOG2392(SERPINB3), KOG3587(LGALS7), KOG3591(CRYAB), KOG0819(ANXA1, 2, 5), KOG3205(ARHGDIB).

As a scaffolding protein, RACK1/GNB2L1 contains two functional domains, namely WD40 repeated protein and β G protein subunit (Figure 5(A)). Through NetPhos2.0 based amino acid phosphorylation prediction, we have mapped the phosphorylation sites of RACK1 in Figure 5(B), consisting 24 possible phosphorylation sites, in which 15 sites are located in serine, 8 sites are located in threonine and 1 site is located in Tyrosine, indicating the cross-interaction with other proteins. The gene-gene interaction network returned by STRING is shown in Figure 5(C), in which RACK1 was overexpressed in OSCC and its two domains (RACK1: COG2319 and KOG0279) serve as key nodes to connect with other deregulated proteins via WD40 repeated protein (number COG2319) and β G protein subunit. WD 40 repeated protein (number COG2319) interacts with 8 differential proteins directly via 15 pathways and β G protein subunit (number KOG0279) directly interacts with 8 differential proteins in 11 interacting pathways. In detail, KOG0279 subunit interacts with EIF5A (KOG2924, KOG0567 and KOG3271), S100A7 (KOG0401), MTPN (KOG0363), S100A8 (KOG1947), UBE2N (KOG0417, COG5078), LGALS7 (KOG3587), SERPINB3 (KOG2392), CSTB (NOG29074) separately. Meanwhile, COG2319 subunit interacts with PRIM2 (COG2219), MTPN (COG0508, COG0666 and COG0459), EIF5A (COG1413, COG1899, COG0231, COG1601 and COG0364), STMN1 (COG2518, NOG75290), UBE2N (COG5078), ANXA1 (p-AKT) (COG5021), SERPINB3(p-AKT, p-mTOR,) (COG4826), TAGLN(AKT pathways) (COG5199) respectively, shaping another network. Notably, we also noticed that most of the RACK1 interacted proteins, such as EIF5A (Ding et al. 2011), CSTB (Zhang, Shi, et al. 2016) and TAGLN (Zhou et al. 2016), are involved with tumor malignancy through PI3 K/pAKT pathways. The above results strongly suggested the crucial role of RACK1 during the progression of OSCC tumorigenesis, which is consistent with other reports (Wang et al. 2009a; Zhang, Liu, et al. 2016).

## Discussion

To the best of our knowledge, this study is one of the few cases which try to decode the expression pattern of OSCC and construct new tumor classifications. Therefore, we have performed the microarray of two pairs of clinical samples of OSCC tumor and adjacent normal tissue to explore the aberrant expression of OSCC related genes and try to depict the gene-gene interaction network. Based on our analysis, we have figured out a number of significantly deregulated genes between OSCC tumor and non-tumor which is potential to link tumor initiation and malignancy. In detail, 3800 genes are up-regulated genes, and 4072 are down-regulated ones. 15 most differential regulated genes between OSCC tumor and non-tumor were further confirmed by both protein expression array and qRT-PCR. RACK1 is among the most overexpressed genes. Gene ontology (GO) analysis has revealed that these aberrant transcripts participate in a series of tumor-related distribution, including metabolic process, cytokine transduction, and cell death regulation. KEGG signaling pathway enrichment suggested a number of pathways (metabolic process and immune response) which are frequently enrolled during cancer progression.

A network graph is constructed based on the interaction of these 15 differential genes by integrated expression signature analysis, including gene expression pattern, GO analysis, KEGG signaling pathway and STRING protein–protein interaction network (Harris et al. 2008), depicting the direct interaction of two subunits of RACK1 with 14 differential proteins in 26 interaction pathways. Thus, it indicates that RACK1 is the core protein of the entire network as well as the key node protein in OSCC, which is consistent with other previous reports that RACK1 is always overexpressed in OSCC and could serve a potential diagnosis marker for patient poor survival (Wang et al. 2009a; Zhang, Liu, et al. 2016). However, few systematic studies have focused on the role of RACK1 in the gene interaction network. Hence we try to explore the underlying mechanism RACK1 in promoting OSCC progression in the context of whole transcription pattern by microarray.

RACK1 is located in the 5q35.3, proximal to the chromosome telomeres. As a scaffolding molecule, RACK1 attracts substantial academic concern by its function as protein kinase receptor, which can serve as the molecular network center by attracting numerous kinases and receptors along with mediate various intracellular signaling pathways (Chang et al. 1998; Liliental and Chang 1998; Yarwood et al. 1999; Hermanto et al. 2002; McCahill et al. 2002; Nilsson et al. 2004; Liu et al. 2007). Scholars have formed a common belief that that RACK1 plays a major role in regulating some important biology process such as cell multiplication, neural system development, metabolism, tumor metastasis, and invasion. Relations among RACK1, neoplasm and metastasis is a hotspot in recent tumor research, like recently reported by Li et al., RACK1 is a key regulator of the cell migration, invasion and adhesion in OSCC (Li et al. 2012). Additionally, expression of RACK1 is an excellent predictor of poor clinical outcome in OSCC as well as other common cancers (Wang et al. 2009b; Cao et al. 2010).

Here DNA expression microarray is exploited to get the overall expression profile of OSCC, together with identifying potential gene candidates related to the incidence and development of OSCC. We found a large proportion of genes were correlated with cancer-related gene ontology and pathways. Moreover, gene interaction network construction further supported the key node of RACK1 in contributing to OSCC carcinogenesis by interaction with other coding genes. Altogether, this systematic analysis of gene expression profile will benefit the optimization of treatment management and provide insights in genome-mining based clinical trials for the serious OSCC patients.
